# Relationships of Early Parenteral Nutrition and Human Milk Fortification on the Growth of Neonates With Birth Weights Between 1500 and 1750 g

**DOI:** 10.1111/jpc.70426

**Published:** 2026-05-17

**Authors:** Yutaro Noguchi, Masahiko Murase, Gakuto Ujiie, Yoko Mizukoshi, Hideyuki Asai, Mio Igawa, Hirokazu Ikeda

**Affiliations:** ^1^ Children's Medical Center, Showa Medical University Northern Yokohama Hospital Yokohama Kanagawa Japan

**Keywords:** early parenteral nutrition, human milk fortification, low birthweight infant

## Abstract

**Introduction:**

Low‐birthweight infants have high nutrient requirements; however, early parenteral nutrition (EPN) and human milk fortification (HF) are provided in very low‐birthweight infants. We changed the indications for EPN and HF from very low birth weight to birth weight < 1750 g in our hospital in April 2019.

**Aim:**

This study aimed to assess the growth and neurodevelopmental relationships in infants with birth weights between 1500 and 1750 g, with or without EPN and HF.

**Methods:**

A retrospective cohort study was conducted using the medical records of infants with birth weights between 1500 and 1750 g from April 2016 to March 2022, who were divided into two groups: before and after the change in indication.

**Results:**

EPN and HF promoted head circumference expansion during hospitalization. However, no relationships on growth were noted after hospital discharge. Furthermore, no difference in the incidence of complications was observed among the preterm infants, and relatively few adverse events associated with EPN and HF led to safe nutritional management.

**Conclusion:**

For infants with birth weights between 1500 and 1750 g, EPN and HF may improve short‐term growth, but may not have an impact in the long term.

## 1 Introduction

Appropriate growth is critical in preterm infants because growth failure is often associated with neurodevelopmental delays [1, 2]. Appropriate growth of preterm infants is suggested by some to be equivalent to foetal growth [1, 2]. To achieve this growth, preterm infants require early parenteral nutrition (EPN) and human milk fortification (HF) to meet their nutritional needs, as they have greater nutritional requirements than full‐term infants to support foetal growth [2].

EPN is a method of initiating parenteral nutrition as soon as possible following birth to provide adequate nutrition. EPN supplements nutrient requirements until enteral nutrition is established, thereby minimizing interruptions in nutritional supply and preventing nutritional crises. Consequently, the growth and development of preterm infants can be accelerated and the incidence of complications can be reduced [1, 3].

Although human milk is the best nutrition source for preterm infants [4, 5], it cannot provide adequate nutrition for preterm infants [1, 6]. HF is widely used to obtain adequate nutrition for preterm infants and is the addition of multiple nutrients to human milk. Preterm infants who received a moderate amount of protein demonstrated better weight and height gain than those who received small protein amounts [7].

Therefore, protein intake is a primary nutritional factor for preterm infant growth. Very low birth weight and low birth weight infants need to meet higher nutritional requirements than full‐term infants. However, as EPN and HF are generally reserved for very low birth weight infants, despite their theoretical benefits, whether EPN and HF are effective for infants weighing < 1750 g remains unclear. We assumed that the neurodevelopment and growth of preterm infants would improve because of EPN and HF. Consequently, we changed the indications for EPN and HF from very low birth weight to birth weight < 1750 g in our hospital in April 2019.

Herein, we reported the short‐ and long‐term effects of EPN and HF in infants with birth weights between 1500 and 1750 g. Our study aimed to assess the growth and neurodevelopmental differences in infants with birth weights between 1500 and 1750 g, with or without parenteral nutrition and HF.

## 2 Methods

### 2.1 Study Design

We conducted a retrospective cohort study using patient medical records. Eligible cases were infants born at our hospital between April 2016 and March 2022 with birth weights of 1500–1750 g. Patients were divided into pre‐intervention (pre‐IG) and post‐intervention (post‐IG) groups. The pre‐IG included infants born between April 2016 and March 2019 with non‐routine use of parenteral nutrition and HF. The post‐IG consisted of infants born between April 2019 and March 2022, with the routine use of parenteral nutrition and HF.

### 2.2 Intervention Methods

EPN was initiated immediately after birth, with various formulations including amino acids, fats and vitamins being added gradually. Amino acids are started at 2 g/kg/day and titrated up to 3 g/kg/day. For HF, we used commercially available HMS‐1 and HMS‐2 (Meiji Co. Ltd., Tokyo, Japan). We added these to breast milk or donor milk once the enteral feeding volume exceeded 50 mL/kg/day. We started with half the recommended concentration, confirmed there were no side effects and then switched to the full recommended concentration a few days later.

### 2.3 Exclusions

The exclusion criteria were as follows: congenital heart disease, surgical treatment, multiple births and transfer to other hospitals.

### 2.4 Patient Characteristics

Gestational age, sex, Apgar score, singleton or multiple births, physical measurements at birth (weight, height, head circumference), length of hospital stay, presence of SGA and extrauterine growth restriction (EUGR), and incidence of complications [respiratory distress syndrome (RDS), chronic lung disease (CLD), intraventricular haemorrhage, sepsis, retinopathy of prematurity (ROP) and necrotizing enterocolitis (NEC), including both medically and surgically managed cases]. EPN‐related adverse events such as chylothorax and catheter infection. Definitions of terms; SGA: Both height‐for‐gestational‐age and weight‐for‐gestational‐age percentiles are below the 10th percentile, RDS: Clinically diagnosed and treated with surfactant, CLD: Requiring oxygen supplementation exceeding 21% at 28 days of age, IVH: Evidence of haemorrhage on head MRI, Sepsis: Cases with positive blood cultures, ROP: Cases that underwent ophthalmic treatment, PDA: Requiring medication or surgery for treatment, Adverse events: Cases requiring therapeutic intervention due to chylothorax or catheter infection during PI catheter insertion.

### 2.5 Maternal Characteristics

Pregnancy and delivery history, and presence of pregnancy complications (gestational hypertension, gestational diabetes, premature rupture of membranes and chorioamnionitis).

### 2.6 Nutritional Characteristics

EPN and HF duration, minimum weight and maximum weight loss, enteral feeding initiation on the day of birth, achievement of 100 mL/kg/day and full feeding (140 mL/kg/day) on the day of birth.

### 2.7 Anthropometric Data

Weight, height and head circumference at 40 weeks of corrected gestational age (CGA) or discharge and at 4, 7, 12 and 18 months of CGA; standard deviation (SD) changes during hospitalization; weight gain (g/day); and growth velocity (GV). SD score for birth, 40 weeks of CGA or discharge and at 4, 7, 12 and 18 months of CGA were calculated using Japanese growth charts [8, 9]. EUGR was defined as a decrease in SD of 1.0 or more in physical measurements from birth to 40 weeks of CGA or discharge [10]. The GV was evaluated using the formula in the previous report [11].

### 2.8 Study Variables

We extracted relevant information from the hospital medical records. Cases with missing outcome data were excluded from the corresponding analyses, and analyses were performed using available data only.

### 2.9 Data Analysis

Continuous variables were presented as medians with interquartile range. Given the small sample size, non‐parametric statistical methods were applied. Differences between the two groups were analysed using the Wilcoxon rank‐sum test for continuous variables. Categorical variables are expressed as numbers and percentages and were compared using Fisher's exact test. Statistical analyses were performed using JMP version 19 (SAS Institute Inc., Cary, NC, USA). A two‐sided *p* value < 0.05 was considered statistically significant. This study was approved by the Ethics Committee on Research Involving Human Subjects at Showa Medical University (Approval No. 2023‐028‐B). Given the retrospective nature of the study, informed consent was obtained using an opt‐out approach.

## 3 Results

### 3.1 Cases Analysed

**FIGURE 1:**
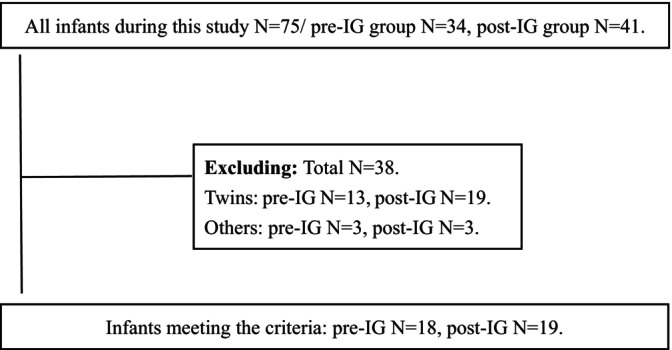
Flow diagram of eligible infants. Eligible infants were born at our hospital between April 2016 and March 2022, with birth weights of 1500–1750 g. The patients were divided into pre‐intervention (pre‐IG) and post‐intervention (post‐IG) groups after April 2019.

Seventy‐five infants were admitted to our hospital between April 2016 and March 2022. In this study, 34 and 41 patients were included in the pre‐IG and post‐IG periods, respectively. Sixteen pre‐IG and 22 post‐IG cases were excluded, and 37 cases (18 pre‐IG and 19 post‐IG) were analysed (Figure 1).

### 3.2 Study Characteristics

Table 1 presents the study characteristics. No differences were noted in the participant characteristics. Table 1 shows the nutritional characteristics of both the groups. EPN was performed in 39% of pre‐IG cases and 79% of post‐IG cases. HF was performed in 17% of the pre‐IG cases and 74% of the post‐IG cases. The reasons for the lack of EPN and HF could not be determined from the medical records. The post‐IG group had more cases of EPN and HF than the pre‐IG group. The number of days of enteral feeding initiation did not differ between the pre‐ and post‐IG groups. However, the post‐IG reached 100 mL/kg/day and full enteral feeding significantly later than the pre‐IG.

**TABLE 1:** Background and characteristics during hospitalization.

	Pre‐IG (*n* = 18)	Post‐IG (*n* = 19)	*p*
Patient characteristics			
**Male**	**6 (33%)**	**13 (68%)**	**0.03**
Gestational age, median (IQR) (weeks)	32.6 (31.8, 34.3)	33.4 (31.7, 35.0)	1.00
Apgar score, median (IQR)			
1 min	8 (4, 8)	6 (4, 8)	0.90
5 min	9 (8, 9)	9 (8, 9)	0.83
Birth anthropometric data, median (IQR)			
Birth weight (g)	1661 (1563, 1705)	1633 (1552, 1696)	0.37
Birth weight (SD)	−0.52 (−1.77, −0.29)	−1.05 (−1.62, −0.37)	0.39
Birth body length (cm)	41.1 (39.8, 42.2)	41.0 (40.0, 43.0)	0.57
Birth body length (SD)	−0.83 (−1.65, −0.01)	−0.62 (−1.21, −0.27)	0.96
Birth head circumference (cm)	30.0 (29.0, 30.4)	29.0 (28.5, 29.8)	0.06
Birth head circumference (SD)	−0.22 (−0.64, 0.43)	−0.74 (−1.38, 0.13)	0.08
SGA	5 (28%)	3 (16%)	0.38
Maternal Characteristics			
Primipara	13 (72%)	12 (60%)	0.42
Gestational diabetes mellitus	3 (17%)	1 (5.3%)	0.26
Hypertensive disorders of pregnancy	6 (33%)	5 (26%)	0.64
Chorioamnionitis	2 (11%)	0 (0%)	0.14
Premature rupture of membranes	5 (28%)	6 (32%)	0.80
Nutritional status, median (IQR)			
**EPN provided case**	**7 (39%)**	**15 (79%)**	**0.01**
**Duration of EPN administration (days)**	**0 (0, 6)**	**8 (4, 11)**	**0.005**
**HF provided case**	**3 (17%)**	**14 (74%)**	**< 0.001**
**Duration of HF administration (days)**	**0 (0, 0)**	**15 (0, 30)**	**0.001**
Maximum percent of weight loss (%)	7.4 (5.8, 9.2)	6.7 (4.2, 9.8)	0.48
Time to regain birthweight (days)	9 (8, 12)	8 (6, 11)	0.19
The initiation day of enteral feeding (days)	1 (0, 1)	1 (0, 1)	0.66
**Time of enteral feeding increased to 100 mL/kg (days)**	**6 (5, 6)**	**7 (5, 8)**	**0.03**
**Time of enteral feeding increased to 140 mL/kg (days)**	**8 (7, 9)**	**9 (8, 11)**	**0.03**
Hospitalization period (days)	40 (34, 53)	46 (37, 63)	0.39
Complications			
Respiratory distress syndrome	1 (6%)	4 (21%)	0.17
Chronic lung disease	0 (0%)	1 (5%)	0.32
Patent ductus arteriosus ligation	0 (0%)	0 (0%)	
Severe intraventricular haemorrhage	0 (0%)	0 (0%)	
Sepsis	0 (0%)	0 (0%)	
Retinopathy of prematurity	0 (0%)	0 (0%)	
Chylothorax	0 (0%)	0 (0%)	
Catheter infection	1 (6%)	0 (0%)	0.30

*Note:* Values with statistically significant differences are presented in bold.

Abbreviations: EPN: parenteral nutrition, HF: human milk fortification, IG: intervention group, IQR: interquartile range, IVH: intraventricular haemorrhage, SD: standard deviation, SGA: small for gestational age, PDA: patent ductus arteriosus.

### 3.3 Anthropometric Growth Data (During Hospitalization)

Table 2 presents the anthropometric growth data at approximately 40 weeks of corrected gestational age and during hospitalization. Anthropometric data demonstrated no differences between the pre‐ and post‐IGs at 40 weeks of CGA or after discharge. However, the post‐IG group revealed a greater SD change in head circumference from birth to 40 weeks CGA or discharge than the pre‐IG group.

**TABLE 2:** Anthropometric data during hospitalization.

	Pre‐IG (*n* = 18)	Post‐IG (*n* = 19)	*p*
Anthropometric data at 40 weeks of corrected age, median (IQR)			
Body weight (g)	2635 (2393, 2824)	2766 (2540, 3146)	0.17
Body weight (SD)	−0.72 (−1.42, −0.52)	−0.62 (−1.77, −0.13)	0.64
Body length (cm)	47.6 (46.3, 49.3)	48.0 (46.8, 49.0)	0.53
Body length (SD)	−0.61 (−1.23, 0.31)	−0.58 (−1.41, −0.20)	0.94
Head circumference (cm)	33.3 (32.4, 34.7)	34.1 (33.2, 35.0)	0.11
Head circumference (SD)	0.14 (−0.65, 0.96)	0.54 (−0.17, 1.26)	0.22
EUGR (body weight)	0 (0%)	0 (0%)	
EUGR (body length)	1 (5.6%)	2 (10.5%)	0.58
EUGR (head circumference)	0 (0%)	0 (0%)	
SD change (body weight)	−0.16 (−0.36, 0.28)	0.18 (−0.15, 0.65)	0.08
SD change (body length)	0.38 (−0.16, 0.55)	−0.005 (−0.33, 0.55)	0.35
**SD change (head circumference)**	**0.35 (−0.41, 0.84)**	**1.30 (0.63, 1.73)**	**0.002**
Weight gain from birth (g/day)	21.5 (19.1, 26.9)	24.9 (21.2, 27.1)	0.21
Weight gain from birth weight regression (g/day)	29.1 (24.3, 34.6)	31.0 (25.4, 34.1)	0.84
GV from birth	10.3 (9.1, 12.1)	11.1 (9.7, 12.5)	0.40
GV from birth weight regression	14.0 (12.2, 15.6)	14.2 (11.8, 15.2)	0.82

*Note:* Values with statistically significant differences are presented in bold.

Abbreviations: EUGR: extrauterine growth restriction, GV: growth velocity, IG: intervention group, IQR: interquartile range, SD: standard deviation.

### 3.4 Complications

Table 1 shows the incidences of complications and EPN‐related adverse events. None of the patients had patent ductus arteriosus, intraventricular haemorrhage, sepsis, or ROP. The incidence of RDS‐, CLD‐ and EPN‐related adverse events did not differ between the pre‐ and post‐IG groups.

### 3.5 Anthropometric Growth Data (After Hospital Discharge)

Table 3 showed the findings of the comparison of anthropometric data after hospital discharge. Body weight at the modified 12‐month time point was greater in the post‐IG and did not differ in other physical measurements. The SD scores for body weight at 12 months were greater in the post‐IG and did not differ between the pre‐ and post‐IG groups at any other time point.

**TABLE 3:** Anthropometric data after discharge from the hospital.

	Pre‐IG (*n* = 18)	Post‐IG (*n* = 19)	*p*
Anthropometric data after discharge, median (IQR)			
4 months of corrected age	*n* = 13	*n* = 14	
Body weight (g)	6250 (5319, 7325)	6192 (5700, 6700)	0.64
Body weight (SD)	−1.16 (−2.15, −0.45)	−1.37 (−1.97, −0.89)	0.34
Body length (cm)	61.3 (59.6, 64.5)	61.4 (59.5, 62.2)	0.87
Body length (SD)	−1.47 (−2.36, −1.35)	−1.90 (−2.50, −1.47)	0.23
Head circumference (cm)	42.0 (40.2, 43.0)	41.1 (40.3, 42.9)	0.79
Head circumference (SD)	0.30 (−0.20, 1.1)	0.10 (−0.70, 1.1)	0.72
7 months of corrected age	*n* = 14	*n* = 16	
Body weight (g)	7425 (6837, 8156)	7573 (7180, 8305)	0.55
Body weight (SD)	−1.04 (−1.64, −0.42)	−0.95 (−1.41, −0.02)	0.60
Body length (cm)	68.0 (64.5, 69.6)	67.4 (64.6, 70)	0.97
Body length (SD)	−1.06 (−1.74, −0.42)	−1.39 (−1.88, −0.42)	0.68
Head circumference (cm)	44.0 (43.0, 44.4)	44.0 (42.7, 45.7)	0.84
Head circumference (SD)	0.40 (−0.25, 0.63)	0.15 (−0.48, 1.4)	1.00
12 months of corrected age	*n* = 9	*n* = 13	
**Body weight (g)**	**8474 (7047, 8621)**	**8942 (8740, 9461)**	**0.01**
**Body weight (SD)**	**−1.27 (−2.42, −0.72)**	**−0.57 (−1.06, −0.25)**	**0.045**
Body length (cm)	73.2 (69.6, 74.7)	73.8 (71.9, 75.1)	0.40
Body length (SD)	−1.28 (−1.95, −0.59)	−1.08 (−1.59, −0.53)	0.74
Head circumference (cm)	45.2 (44.2, 46.1)	45.6 (44.5, 48.0)	0.26
Head circumference (SD)	−0.2 (−0.5, 0.2)	−0.1 (−0.75, 1.15)	0.71
18 months of corrected age	*n* = 8	*n* = 12	
Body weight (g)	9547 (8087, 10 474)	9765 (8887, 10 432)	0.62
Body weight (SD)	−1.14 (−2.18, −0.25)	−0.71 (−1.23, −0.45)	0.67
Body length (cm)	79.4 (75.3, 81.4)	77.4 (75.0, 80.3)	0.56
Body length (SD)	−0.71 (−1.74, −0.15)	−1.60 (−1.89, −0.55)	0.35
Head circumference (cm)	47.0 (45.6, 47.9)	47.2 (46.3, 48.2)	0.44
Head circumference (SD)	−0.2 (−1.0, 4.5)	−0.15 (−0.45, 0.55)	0.46

*Note:* Values with statistically significant differences are presented in bold.

Abbreviations: IG: intervention group, IQR: interquartile range, SD: standard deviation.

### 3.6 Nutrition Management Policy and Actual Management

Proportions of 39% and 17% of infants in the pre‐IG group underwent EPN and HF, respectively; 21% and 26% of infants in the post‐IG did not undergo EPN and HF, respectively. These percentages were high, and we assumed that the data from these participants influenced our results.

### 3.7 Analysis Limited to Cases That Complied With the Nutritional Management Guidelines

Thus, we excluded the data of these participants and compared the pre‐ and post‐IG values as a secondary analysis. The SD change in body weight and head circumference from pre‐ to post‐IG significantly increased from −0.26 SD to +0.03 SD (*p* = 0.02) and from −0.05 SD to +1.53 SD (*p* < 0.01), respectively. However, no differences were noted in other anthropometric data between the pre‐ and post‐IG groups.

## 4 Discussion

The post‐IG group showed a substantially greater increase in head circumference than the pre‐IG group. Growth in head circumference is clinically crucial because it is closely linked to neurodevelopmental outcomes [12, 13]. The previous meta‐analysis revealed that increased protein administration substantially improved body weight and head circumference [14]. However, this meta‐analysis mainly focused on studies conducted in very low birth weight (VLBW) infants. Can et al. [15] revealed that parenteral protein administration in high doses improved head circumference and height measurements at 40 weeks of CGA compared to standard‐dose parenteral protein administration in preterm infants between 27 and 33 weeks of CGA. Although this study focused on more premature participants than ours did, it showed that adequate protein is necessary for head circumference growth. Therefore, augmented protein intake due to EPN and HF may have contributed to head circumference growth in preterm infants weighing 1500–1750 g in our study.

Our study showed no significant difference in weight gain pre‐ and post‐IG. Adequate protein intake improves body weight growth [14, 15]. Since participants with EPN and HF were included in the pre‐IG group, and those without EPN and HF were included in the post‐IG group, these factors may have affected the results. We performed a second analysis that excluded participants with EPN and HF from the pre‐IG and those without EPN or HF from the post‐IG. The post‐IG group had a substantially greater weight gain than the pre‐IG group. Therefore, the augmented protein intake owing to EPN and HF contributed to body weight growth in preterm infants weighing 1500–1750 g. Our study supported the criticality of adequate protein intake for improving weight and head circumference growth, even in infants with low birth weight.

Our study revealed no difference in length growth between pre‐ and post‐IG. However, a meta‐analysis revealed that HF has a growth‐promoting effect on height [7]. This finding was based on a study that primarily focused on VLBW infants. Considering that this study included children with birth weights between 1500 and 1750 g, the participants in the meta‐analysis were more premature than our study participants. Hence, a short HF diet duration may influence height growth. The achievement day of life for 100 mL/kg/day and full feeding in the post‐IG group was delayed compared with that in the pre‐IG group. Although the exact reasons for this were unclear, the factor of the primary physician was considered a potential cause. Given that our institution has not established a standard protocol for the daily increment in feed intake, the decision was made at the attending physician's discretion. Our team members were regularly rotated, which may alter the approach and influence the incremental feeding intake. Furthermore, because EPN and HF were provided to VLBW infants and critical cases before the protocol change, the primary physician may have cautiously increased feeding intake. The early establishment of enteral nutrition reduced EPN‐related adverse events [16, 17]. Variations in incremental feeding intake among primary physicians were likely to be minimized by establishing a standard protocol for daily increments in feeding intake. Therefore, a standard protocol for the daily increment in feed intake is needed for the early establishment of enteral nutrition.

Anthropometric data were compared between pre‐ and post‐IG, from 4 to 18 months, and were examined for long‐term differences. The post‐IG group demonstrated a markedly higher body weight than the pre‐IG group at 12 months corrected age. However, no differences were observed in body weights at the other corrected ages. Although body weight at 12 months was higher in the post‐IG, this was not consistent across time points, and its association with EPN or HF remains uncertain. We reviewed medical records to identify the influence of disease incidence, as well as additional factors contributing to the impact of body weight at 12 months corrected age. No difference in disease incidence was noted following discharge and the information in the medical records did not allow us to determine the cause of the difference in weight at the modified 12‐month follow‐up. Nutritional factors following discharge were suspected of affecting weight at 12 months. However, our study could not demonstrate this relationship because it was retrospective and we did not account for these factors. Therefore, a prospective study focusing on the relationship between nutritional factors after discharge and the growth of premature infants is necessary. EPN in the early postnatal period can decrease the incidence of bronchopulmonary dysplasia, sepsis and ROP [1, 18]. However, in our study, the incidence of complications did not significantly differ between the groups. Infants with a gestational age of 32–36 weeks have a low ROP and sepsis incidence [19]. Our study group consisted of infants whose birth weights were between 1500 and 1750 g, who were considered relatively mature, and therefore less likely to develop severe complications. Therefore, the benefits of reducing complications in infants with birth weights between 1500 and 1750 g were likely limited.

Feed intolerance and necrotizing enterocolitis (NEC) are adverse events linked to HF [20]. None of the participants who used HF experienced feed intolerance. Although infants with NEC were excluded in order to focus on growth‐related outcomes, this could have resulted in selection bias. However, in the study, no cases were excluded due to NEC. Therefore, we performed HF without any adverse events.

EPN and HF improved head circumference and weight gain during hospitalization. Whether EPN or HF had an impact on this finding remains unclear. Brown et al. [14] reported that higher protein intake was associated with improved weight gain and head circumference growth during NICU hospitalization. Therefore, in our study, the increased protein intake achieved through EPN and HF may have contributed to the observed improvements in weight and head circumference. Alexander et al. [21] reported no significant difference in growth among moderate‐to‐late preterm infants receiving additional nutritional support (EPN and/or donor milk or formula before full enteral feeding) compared with those who did not, possibly because the time to reach full enteral feeding was relatively short. Similarly, in our study, the duration of EPN was relatively short; therefore, HF may have contributed more to increased protein intake than EPN. Furthermore, a peripherally inserted central catheter carries an infection risk. Thus, our criteria will be altered from providing EPN and HF to providing HF only for infants weighing 1500–1750 g.

## 5 Limitations

The number of cases compared in this study was 38, a small number which may not have been sufficient for comprehensive research. Additionally, the influence of the factors other than nutritional management could not be assessed because of the difference in timing between the pre‐IG and the second periods. Furthermore, the indications for EPN and HF were simultaneously altered. Thus, we could not determine the strongest influence. Because this was a single‐centre retrospective study with a relatively small sample size, the generalizability of our findings may be limited.

## 6 Conclusion

EPN and HF improved head circumference growth in infants with birth weights ranging from 1500 g to 1750 g during NICU hospitalization; however, no consistent long‐term differences were observed. Thus, EPN and HF may influence short‐term growth, although further studies are needed to clarify potential longer‐term effects.

## Funding

The authors have nothing to report.

## Ethics Statement

This study was approved by the Ethics Committee on Research Involving Human Subjects at Showa Medical University (Approval No. 2023‐028‐B).

## Conflicts of Interest

The authors declare no conflicts of interest.

## Supporting information


**Data S1:** The STROBE reporting checklist.

## Data Availability

The data that support the findings of this study are available from the corresponding author upon reasonable request.
